# Metabolomic analysis of pathways related to rice grain chalkiness by a notched-belly mutant with high occurrence of white-belly grains

**DOI:** 10.1186/s12870-017-0985-7

**Published:** 2017-02-07

**Authors:** Zhaomiao Lin, Xincheng Zhang, Zunxin Wang, Yutong Jiang, Zhenghui Liu, Danny Alexander, Ganghua Li, Shaohua Wang, Yanfeng Ding

**Affiliations:** 1 College of Agronomy, Nanjing Agricultural University/Key Laboratory of Crop Physiology Ecology and Production Management, Ministry of Agriculture, Nanjing, 210095 People’s Republic of China; 2 Crops Research Institute, Fujian Academy of Agricultural Sciences, Fuzhou, 350013 People’s Republic of China; 3 Jiangsu Collaborative Innovation Center for Modern Crop Production, Nanjing, 210095 People’s Republic of China; 4 Metabolon Inc, Durham, NC 27713 USA

**Keywords:** Grain chalkiness, Metabolomic analysis, Notched-belly mutants, Rice, White-belly

## Abstract

**Background:**

Grain chalkiness is a highly undesirable trait deleterious to rice appearance and milling quality. The physiological and molecular foundation of chalkiness formation is still partially understood, because of the complex interactions between multiple genes and growing environments.

**Results:**

We report the untargeted metabolomic analysis of grains from a notched-belly mutant (DY1102) with high percentage of white-belly, which predominantly occurs in the bottom part proximal to the embryo. Metabolites in developing grains were profiled on the composite platforms of UPLC/MS/MS and GC/MS. Sampling times were 5, 10, 15, and 20 days after anthesis, the critical time points for chalkiness formation. A total of 214 metabolites were identified, covering most of the central metabolic pathways and partial secondary pathways including amino acids, carbohydrates, lipids, cofactors, peptides, nucleotides, phytohormones, and secondary metabolites. A comparison of the bottom chalky part and the upper translucent part of developing grains of DY1102 resulted in 180 metabolites related to chalkiness formation.

**Conclusions:**

Generally, in comparison to the translucent upper part, the chalky endosperm had lower levels of metabolites regarding carbon and nitrogen metabolism for synthesis of storage starch and protein, which was accompanied by perturbation of pathways participating in scavenging of reactive oxygen species, osmorugulation, cell wall synthesis, and mineral ion homeostasis. Based on these results, metabolic mechanism of chalkiness formation is discussed, with the role of embryo highlighted.

**Electronic supplementary material:**

The online version of this article (doi:10.1186/s12870-017-0985-7) contains supplementary material, which is available to authorized users.

## Background

Chalkiness is the opaque portion in rice endosperm due to loose packing of starch granules and protein bodies [1, 2]. Depending on its location on or within the endosperm, chalkiness is grouped into white-belly, white-core, white-back, etc. Grain chalkiness is a highly undesirable trait, with detrimental effects on rice appearance quality as well as milling, eating, and cooking quality. High occurrence of grain chalkiness has been a major problem facing many rice-producing areas throughout the world, in particular in a warming climate [3]. Reducing chalky grain rate has been one of the primary objectives for rice breeders and producers.

Chalkiness is a complex quantitative trait, governed by polygenes and subjected to large variations in environmental factors like temperature and humidity and cultural practices such as fertilization and irrigation [4]. By molecular marker-based QTL analyses of numerous mapping populations, more than 140 QTLs were identified across all 12 chromosomes for the chalkiness trait, mostly among Asian cultivars [2]. Of these, several major QTLs such as on chromosomes 1, 5, 7, 8, and 9 are stably expressed across multiple environments or various populations [5]. In addition, several possible candidate genes responsible for grain chalkiness have been cloned by fine mapping, including *pyruvate orthophosphate dikinase*, *starch synthase IIIa*, *UDP-glucose pyrophosphorylase*, *cell wall invertase*, and *H* ^*+*^ *-translocating pyrophosphatase* (V-PPase)*,* as reviewed by Sreenivasulu et al. [2]. Based on this, the molecular basis of chalkiness formation is partly understood. Li et al. [6] studied the function of *Chalk5*, which encodes V-PPase with inorganic pyrophosphate hydrolysis and H^+^-translocation activity. They found that *Chalk5* could disturb pH homeostasis in the endomembrane trafficking system, resulting in an abnormal decrease in protein body number and size, causing aberrant shape and spatial arrangements of starch granules and protein bodies.

Rice quality is essentially determined by chemical composition of the storages of starch and protein that derive from carbon (C) and nitrogen (N) metabolism during grain filling. Metabolomics is one of the “omics” studies for integrative analysis of metabolites in organisms [7]. Together with genomics, transcriptomics, and proteomics, it is a powerful tool to provide a link between genotype and phenotype and to generate new knowledge of systems biology. Besides providing an understanding of the metabolic state in plants under various circumstances, it can be applied to clarify the functions of unknown genes by using natural variants or mutants of the target plants, and might be useful in breeding programs because of the close relation between quality traits like taste and metabolic conditions [8]. In rice, metabolomic approaches have yielded new insights into the composition and regulation of seed metabolism [9], correlations between the metabolic phenotype and geographic origin of japonica and indica rice [10], grain filling-related metabolism under high temperature [11], food quality prediction [12], and the bioactive compounds in cooked rice [13]. However, there is little information concerning the metabolomic analysis of grain chalkiness formation, and the metabolic foundation of it remains unclear. In addition, few studies were conducted to investigate the metabolic changes during the early stage of grain filling, which is the critical time point for chalkiness formation.

Using chemical mutagen of EMS, we indentified a notched-belly mutant with high ratio of white-belly grains [14]. The notched-line divided grains into two parts, with the upper part nearly all translucent whereas the bottom part, proximal to the embryo, having high occurrence of white-belly. Using the matured grains of this mutant, Lin et al. [14] developed a novel comparison system that can minimize the influence of genetic background and growing environment, and conducted a comprehensive survey of endosperm proteomics, unraveling diverse but well regulated pathways responsible for grain chalkiness. In this study, we profiled and compared the metabolomes between the upper translucent part and the bottom chalky part of developing grains from the notched-belly mutant. In total, 214 metabolites were identified, among them the abundances of 180 metabolites were significantly altered in the chalky part. These altered metabolites indicated that chalkiness formation was associated with a metabolic shift from C and N metabolism to reactive oxygen species (ROS) scavenging, osmorugulation, cell wall synthesis, and iron homeostasis. Levels of metabolites related to secondary metabolism and plant hormone biosynthesis were also remarkably altered in the chalky part. These results extend our understanding of metabolomic mechanism underlying grain chalkiness.

## Results

**Fig. 1:**
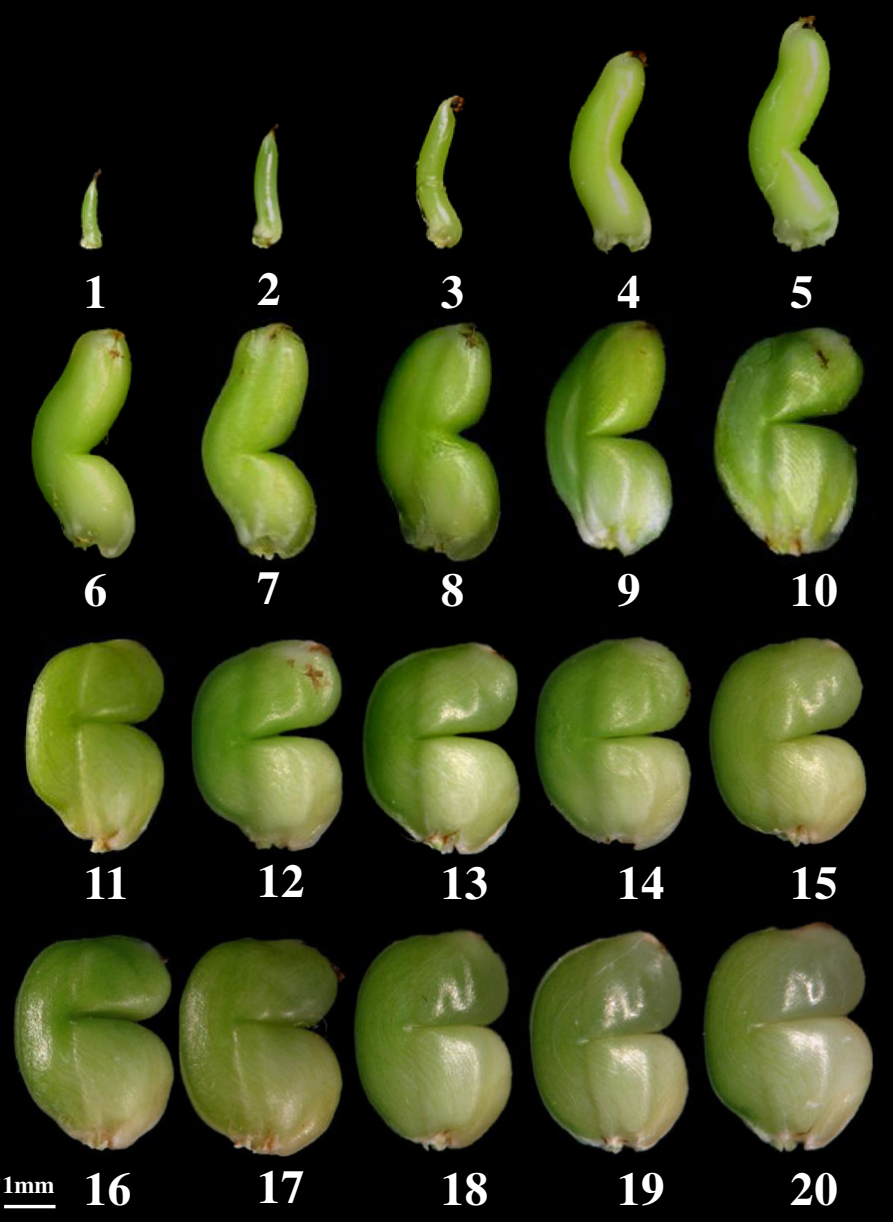
Morphological changes during caryopses development of the notched-belly mutant with high occurrence of white-belly grains. The notched-line is visible on 5 DAA (days after anthesis), and the white-belly occurs on 20 DAA. Sampling times are 5, 10, 15, and 20 DAA, when the grains are cut into two parts (the upper and the bottom part) along the notched-line. *Numbers* below each grain indicate the days after anthesis

We obtained a notched-belly mutant with a high ratio of while-belly grains. The notched line can be seed after 5 DAA, possible due to the restriction of the hulls on caryopsis elongation (Fig. 1). The constriction by bending alters the morphology of the caryopsis, resulting in different shapes between the upper and lower parts, as shown in Fig. 1. It is possible that this change in morphology is directly responsible for the difference in metabolism between the two halves, perhaps due to constriction of metabolite supply form the lower to the upper parts. Indeed, a germination experiment conducted in our lab in 2014 showed that the upper part remained unchanged at 3 weeks after germination, while the lower part near to the embryo was entirely decomposed (data not shown), indicating the substance flow may be prevented by the notched-line. However, the two parts of the grain are thought to mature independently as they are supplied with assimilates from source organs separately through vascular bundles on the dorsal side [15].

### Metabolite profiling of rice endosperm

The notched-belly mutant has high percentage of notched-belly grain, being 66.58% under greenhouse condition in 2014 (Table 1). The notched line at the ventral part is visible on 5 day after anthesis (DAA), and the major part of the notched-belly grains has white-belly, being visible on the 20 DAA (Fig. 1). Notably, the middle primary rachis of the panicle had relatively higher ratio of white-belly grains, being 80.54% in greenhouse in 2014 (Table 1). Moreover, most of the white-belly, 95.62% in greenhouse, occurs in the bottom half part proximal to the embryo, i.e., below the notched line.

**Table 1:** Positional variation of white-belly occurrence within panicle under different growing conditions for the notched-belly mutant

Grain position	Notched-belly grain/total grain (%)			White-belly grain/notched-belly grain (%)		
	AT-2014	GH-2014	HNT-2014	AT-2014	GH-2014	HNT-2014
TPR	64.83	89.29	73.29	99.06 (17.51)	80.79 (2.96)	96.34 (4.88)
TSR	80.39	63.74	76.44	87.35 (6.02)	81.13 (5.66)	88.81 (2.10)
MPR	68.64	78.57	77.07	97.17 (14.62)	83.71 (3.17)	98.99 (6.53)
MSR	80.21	59.87	72.91	84.23 (5.05)	83.87 (4.30)	90.50 (2.07)
BPR	72.14	56.98	79.59	91.41 (14.65)	76.19 (6.80)	92.44 (4.62)
BSR	88.72	42.53	70.18	77.93 (2.70)	82.19 (5.48)	89.68 (1.29)
Whole panicle	75.67	66.58	74.86	89.11 (9.76)	81.52 (4.38)	93.13 (3.76)

Note: Data in the brackets are ratio of grains with white-belly both in the upper and bottom parts

*AT* ambient temperature, *GH* greenhouse, *HNT* high night temperature by reflecting film covering over the canopy in the night, with a 0.8 °C increase. *TPR* top primary rachis, *TSR* top secondary rachis, *MPR* middle primary rachis, *MSR* middle secondary rachis, *BPR* bottom primary rachis, *BSR* bottom secondary rachis

In previous proteomic survey of the mature endosperm of the mutant, Lin et al. [14] found that several key proteins involved in carbohydrate metabolism (especially cell wall synthesis) and protein synthesis, folding and degradation were responsible for rice grain chalkiness. These proteomic data prompted us to investigate the metabolic changes related to chalky tissue formation during grain development.

**Fig. 2:**
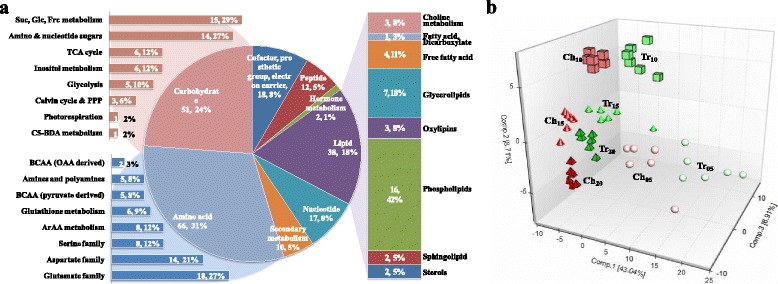
Classification and distribution of the identified metabolites in developing caryopses of the notched-belly mutant (**a**) and the principal component analysis plot (**b**). **a**, Distribution of identified 214 metabolites in pie chart, sub-classifications of amino acids, carbohydrates, and lipids are shown with bar charts and histogram, respectively. **b**, Principal component analysis (PCA) model of eight samples at four developmental time points with six biological replications, where Ch represents the bottom chalky part and Tr the translucent upper part. *ArAAs* Aromatic amino acids, *BCAAs* Branched-chain amino acids, *C5-BDA* C5 branched dibasic acid, *PPP* Pentose phosphate pathway

By subjecting the translucent (Tr) upper part and chalky (Ch) bottom part of developing grains produced in the greenhouse to three analytical platforms (UPLC/MS/MS optimized for basic species, UPLC/MS/MS optimized for acidic species, and GC/MS optimized for small, volatile, and thermally stable molecules), we identified 214 rice grain metabolites (Additional file 1). The 214 metabolites could be assigned into eight super pathways (Fig. 2a) and subsequent 39 sub-pathways (Additional file 1) according to Plant Metabolic Network (PMN) and Kyoto Encyclopedia of Genes and Genomes (KEGG). These metabolites covered mainly the central metabolism pathways and partial secondary metabolism pathways, including 66 amino acids, 51 carbohydrates, 38 lipids, 18 CPGECs (cofactors, prosthetic groups, electron carriers), 17 nucleotides, 12 peptides, 10 secondary metabolites, and 2 phytohormones. The metabolome list of this study is similar to those identified for maize kernel [16], and rice seed [10] and anther [7]. A non-supervised principal component analysis (PCA) was performed with all 214 metabolites. As shown in Fig. 2b, there was a clear separation between Ch and Tr parts at all developmental time points, indicating that the metabolom of chalky endosperm was distinct from that of translucent endosperm. The tight groupings of most of the groups also demonstrate the good biological reproducibility within groups.

### Metabolic changes regarding developmental stages

During rice seed development, the embryo and the endosperm develop synchronously in terms of cell division and differentiation, and complete their morphological differentiation and development on about 10 DAA [17]. After this, embryo tissues ripen physiologically, and the endosperm accumulated the majority of its storage substance on 25 to 30 DAA. Notably, the formation of chalkiness starts on about 5 DAA, as reported by Li et al. [6] who showed that *Chalk5* expression levels were much higher on 5 and 10 DAA.

**Fig. 3:**
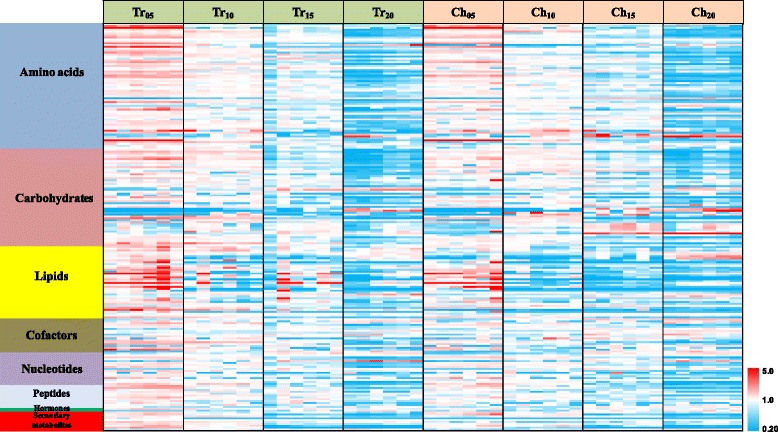
Heat map presentation of the variation of the 214 metabolites with developmental stages. Each *line* in this heat map represents a metabolite. The value of each compound indicates the relative content that were normalized directly on a similar graphical scale, and scaled by their median values for each compound. *Red* indicates high content and *blue* low content of metabolite in the sample

We investigated the dynamics of metabolomic changes in translucent and chalky endosperm with a interval of five days through seed development, i.e., 5, 10, 15, and 20 DAA. Statistical tests compared various time-related changes within each part, as well as compared the two parts to each other at each developmental time point. Six biological replicates were provided for each test group. A heat map display of all data showed a clear overall decline in relative levels of the vast majority of compounds as seeds matured (Fig. 3). This is the expected pattern as carbon and nitrogen are incorporated into macromolecules (starch, proteins, and lipids) as the seeds approach the quiescent state of maturation. The map also shows the generally good biological reproducibility within the groups.

The magnitude of biological differences, whether observed over developmental time, or between the two parts, can be judged by a simple summarization of compounds which met the statistical cut-off criterion (*p* ≤ 0.05). Note that in the time course comparisons there were many more compounds which decreased over time, as opposed to increasing over time (Table 2), consistent with the observation of the heat map above. Because the translucent Tr part was designated as the control in the pair-wise comparisons with the chalky Ch part, we calculated the changes as the ratio of Ch/Tr in all cases. That is, compounds with increased levels were higher in Ch part, the chalky endosperm, while those with decreased levels were higher in Tr, the translucent part. By inspecting these numbers, Tr part had many more compounds which were significantly higher on 5 DAA, but on 10 and 15 DAA this trend was weakening, and by the final time point (20 DAA) there were more compounds which were higher in the Ch part. This suggests significantly different dynamics for endosperm development in the two parts.

**Table 2:** Number of the significantly altered metabolites in relation to developmental and chalkiness effects (*p* ≤ 0.05)

Ratio	Total number of the significantly changed	Number of increase	Number of decrease
Developmental effects			
Tr_10_/Tr_05_	119	23	96
Tr_15_/Tr_05_	150	23	127
Tr_20_/Tr_05_	180	26	154
Ch_10_/Ch_05_	122	31	91
Ch_15_/Ch_05_	157	46	111
Ch_20_/Ch_05_	181	44	137
Chalkiness effects			
Ch_05_/Tr_05_	71	5	66
Ch_10_/Tr_10_	61	13	48
Ch_15_/Tr_15_	99	42	57
Ch_20_/Tr_20_	75	42	33

*Ch* the chalky bottom part, *Tr* the translucent upper part

### Altered amino acid metabolism in the chalky endosperm

**Fig. 4:**
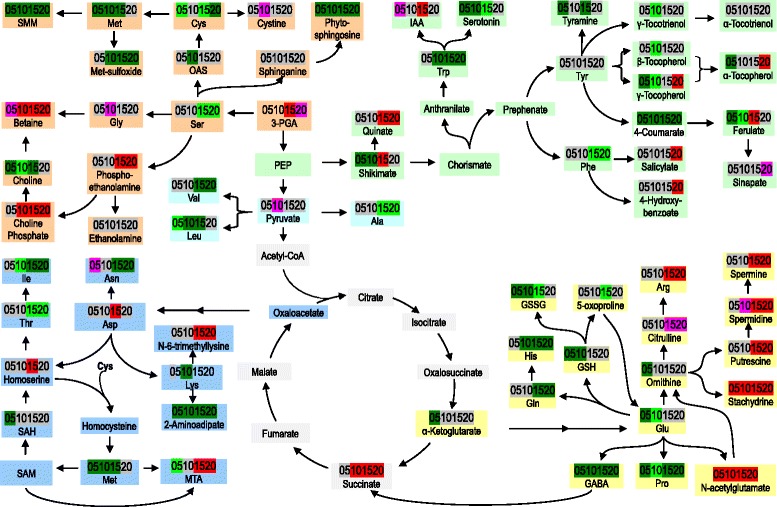
Amino acids and their derivatives of the upper translucent part and the bottom chalky part. 05, 10, 15, and 20, days after anthesis. *Red* and *green* indicate *p* ≤ 0.05 (*red* denotes significant increase in the chalky endosperm, *green* significant decrease). *Pink red* and *light green* indicate 0.05 < p < 0.10 (*pink red* indicates increase while *light green* decrease). Refer to Additional file 1: Table S1 for detailed information of the changes of these metabolites

Except for Gly, Asp, and Arg, chalky parts contained lower levels of amino acids than translucent parts (Fig. 4). The reduction in amino acids coincided with alternation (basically increase) in their derivatives which function as osmolytes and antioxidants.

#### Glutamate-derived amino acids

Amino acids Glu, Gln, His, and Pro were decreased in the chalky endosperm, while Arg was up-regulated (Fig. 4). The three common polyamines in plants, putrescine (Put), spermidine (Spd), and spermine (Spm) were all elevated since 10 DAA. Stachydrine (proline betaine) was increased during all the four sampling times. The acetylation of glutamate into N-acetylglutamate (NAG) is the first step of conversion of Glu to Arg. The higher level of NAG was accompanied by higher levels of citrulline and Arg on 15 DAA and 20 DAA, despite of reduction in ornithine on 5 DAA. The chalky endosperm also had lower levels of γ-aminobutyric acid (GABA), but this compound declined in both parts over time, reaching its minimum on 15 DAA. In addition, chalky endosperm showed lower levels of oxidized glutathione (GSSG) in the mid-development time points, suggesting a less oxidative environment. Consistent with this, there were lower levels of reduced glutathione (GSH) and 5-oxoproline (5-OP).

#### Aspartate-derived amino acids

Generally, amino acids Asn, Ile, Thr, and Lys showed a decreasing trend in the chalky endosperm, while Asp was increased on 15 DAA and Asn slightly increased on 10 DAA as well (Fig. 4). In addition, 2-aminoadipate, derived from Lys, was decreased over time. N-6-trimethyllysine, a protein-lysine degradation product, was significantly higher in chalky endosperm at the late stages of sampling (15 and 20 DAA).

#### Serine-derived amino acids

**Fig. 5:**
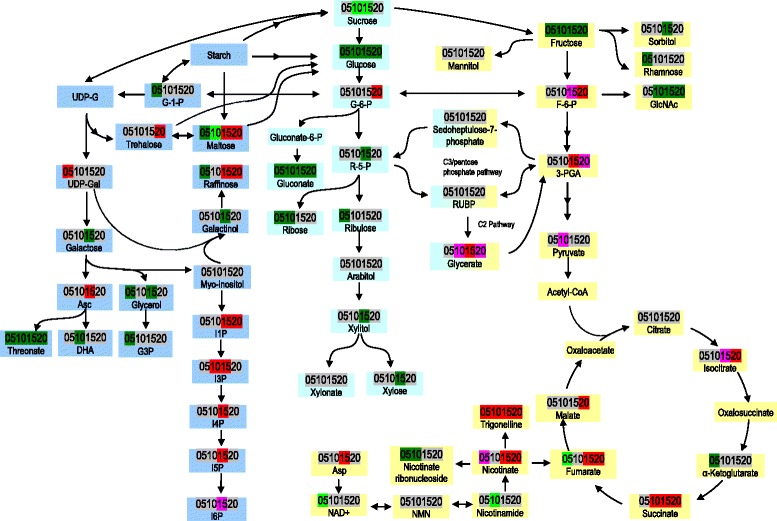
Carbohydrates and their derivatives of the upper translucent part and the bottom chalky part. 05, 10, 15, and 20, days after anthesis. Refer to Additional file 1: Table S1 for detailed information of the changes of these metabolites. Note that G-6-P, the other precursor of I1P, was not presented in this figure. “” indicates multiple steps of reactions

Ser was reduced on 15 DAA and 20 DAA, whereas Gly was increased slightly on 10 DAA in the chalky endosperm (Fig. 4). One of the most consistent and strongest effects observed is the tendency for chalky endosperm to harbor higher levels of several tertiary amino compounds (betaines), which are strong osmolytes. These included trigonelline (nicotinate betaine; Fig. 5), betaine (glycine betaine), stachydrine, phosphocholine (emanating from lipid membrane synthesis or degradation), and N-6-trimethyllysine (Fig. 4).

#### Aromatic amino acids

The aromatic amino acids, Phe, Tyr, and Trp, are central molecules in plant metabolism, serving as precursors for a wide range of secondary metabolites. Their precursor, shikimate, was decreased on 5 DAA and 10 DAA, but increased on 15 DAA (Fig. 4). Trp and Phe showed a decreasing trend, while Tyr had no clear trend. By contrast, the derivative of Tyr, tyramine, was reduced. Serotonin is thought to serve antioxidant and defense functions in plants. It was detected to decrease in the chalky endosperm from to 5 DAA to 15 DAA. In addition, quinate, a metabolite synthesized in a lateral branch of the shikimate biosynthesis pathway, was increased on 10 DAA and 15 DAA. Although its physiological role has not been completely elucidated, quinate accumulation has been detected in plants during fungal invasion [18] and after treatment with herbicides that inhibit amino acid biosynthesis [19].

#### Sulfur-containing amino acids

Sulfur is an essential macronutrient, and is primarily used to synthesize Cys, Met, and numerous essential and secondary metabolites, such as 5′-methylthioadenosine (MTA), S-methylmethionine (SMM), and *S*-adenosylmethionine (SAM). Cys, Met, and SMM were reduced in the chalky endosperm, while MTA showed an increasing trend irrespective of a decrease on 5 DAA (Fig. 4). Methionine sulfoxide (MetO), the product of Met oxidation by reactive oxygen species, was reduced. Similarly, S-adenosyl-homocysteine (SAH), the intermediate involving in the metabolic regeneration of SAM through the SAM cycle, was also declined. In addition, O-acetylserine (OAS), the intermediate of the pathway of Ser to Cys, was decreased on 10 DAA.

### Altered C metabolism in the chalky endosperm

One important metabolite pool in plants is the hexose phosphates, and another is composed of pentose phosphate pathway intermediates and the triose phosphates glyceraldehydes 3-phosphate and dihydroxyacetone phosphate [20]. The hexose phosphate pool consists of three interconvertible metabolic intermediates: glucose 6-phosphate (G6P), glucose 1-phosphate (G1P), and fructose 6-phosphate (F6P), deriving from phosphorylation of free hexose or degradation of starch and sucrose. The hexose pool contributes intermediates to glycolysis as well as many biosynthetic processes including starch and sucrose synthesis, cell wall formation, and the oxidative reactions of the pentose.

#### F6P and its derivatives

F6P and intermediates in glycolysis pathway and tricarboxylic acid (TCA) cycle were altered in the chalky endosperm. F6P and 3-PGA in the chalky endosperm showed a similar trend, being not significantly different on 5 DAA and 10 DAA, but higher on 15 DAA and 20 DAA in comparison with that in the translucent endosperm (Fig. 5). Pyruvate increased slightly only on 10 DAA in the chalky endosperm. In TCA cycle, four metabolites, malate, fumarate, succinate, and isocitrate, were elevated at early maturation stage of 15 DAA or 20 DAA, whereas α-ketoglutarate was reduced on 5 DAA. Generally, these intermediates demonstrated an increasing pattern, indicated that in the chalky endosperm, glycolysis and TCA cycle was shifted to mainly produce selective intermediates involved in metabolism of amino acids, fatty acids, secondary metabolites, and cell wall.

#### G6P and its derivatives

In the chalky endosperm, G6P showed no significant variation from 5 DAA to 15 DAA, but increased significantly on 20 DAA (Fig. 5). It was worth noting that significantly increased G6P was accompanied by significantly decreased glucose. Glucose, the precursor of G6P and cell wall biosynthesis, was reduced during all the four sampling times. In addition, abundances of other plant cell wall precursors, including gluconate and xylose, showed the similar decreasing trend. On the other hand, arabitol did not change markedly in the chalky endosperm. In the pentose phosphate pathway, ribose 5-phosphate was significantly decreased on 15 DAA, while sedoheptulose-7-phosphate was not significant altered. Glycerate involving in photorespiration tended to be increased, slightly on 10 DAA and 20 DAA and markedly on 15 DAA. Furthermore, ribose and ribulose, the crucial components of nucleic acids, were decreased in the chalky endosperm.

#### G1P and its derivatives

G1P in the chalky endosperm was reduced on the beginning of cell division (5 DAA), and then kept at a comparable level after that (Fig. 5). Sucrose, the main C-transport substance from leaf to grain, was decreased on 10 DAA and 15 DAA, indicating the insufficient supply of photosynthetic assimilates of the source organs. Albeit there were significant increases of maltose, it was coupled with decrease in glucose and fructose, implying an accelerated breakdown of starch or a retarded conversion of maltose to glucose and fructose.

Chalky endosperm accumulated significantly higher levels of raffinose than translucent endosperm (Fig. 5). UDP-galactose, one of the precursors of raffinose, was also higher at most time points, although galactinol, its direct precursor, was similar in the two parts at most time points. Consistent with the increase in raffinose, two sugar alcohols, erythritol and threitol, also showed this pattern (Additional file 1).

In addition to higher raffinose, chalky endosperm also showed higher starch intermediates and hexose phosphates than translucent endosperm in the later time points (Additional file 1). The translucent part had higher levels of sucrose and hexoses in all time points except for the final one. Whether this pattern suggests different qualitative mechanisms for regulating carbon distribution in these lines, or if it only reflects slightly shifted kinetics, is not known.

Both kinds of endosperms accumulated phytic acid (inositol 6-phosphate, I6P) and its precursor I5P over the time course, with the trend accelerated on 20 DAA (Fig. 5). However, chalky endosperm showed slightly more phytic acid, and significantly more I5P in the last two time points. I3P was somewhat higher in chalky tissue only at the intermediate time points, while I1P was similar to I5P in its pattern, except that these compounds did not change in translucent endosperm over development. It is possible that early steps in phytate synthesis are accelerated in chalky endosperm, thus pushing the reactions; alternatively, slower conversion to the final product phytic acid in chalky endosperm might account for higher levels of intermediates.

For precursors of cell wall, rhamnose was decreased on 5 DAA, while arabinose did not varied significantly (Fig. 5). Furthermore, trehalose, an alpha,alpha-1,1-linked glucose disaccharide which is indispensable for regulating sugar utilization, was elevated on 20 DAA. It is worth noting that there was a co-occurring decrease of glycerol and glycerol-3-phophosphate (G3P), both of which are associated with lipid metabolism.

### Other metabolites related to chalky endosperm formation

#### Lipids

In general, most of lipids showed a decreasing trend in the chalky tissue (Additional file 1). However, it was noted a somewhat different kinetic pattern for developmental-related changes in several lipids. Four free fatty acids including eicosenoate, linoleate, linolenate, and oleate were all reduced in the chalky part. Similarly, glycerolipids like 1-linoleoylglycerol and 2-linoleoylglycerol, phospholipids such as 1-oleoylgly- cerophosphocholine and glycerophosphorylcholine, and phytosterols of beta-sitosterol and campesterol as well as their precursor squalene, had reduced level in the chalky endosperm. Phytosphingosine was low in chalky endosperm throughout development, but was high in translucent part early, followed by a decline. Its precursor sphinganine was similar for both parts. Notably, in choline pathway, choline was decreased, while its precursors choline phosphate and phosphoethanolamine were increased (Fig. 4).

**Fig. 6:**
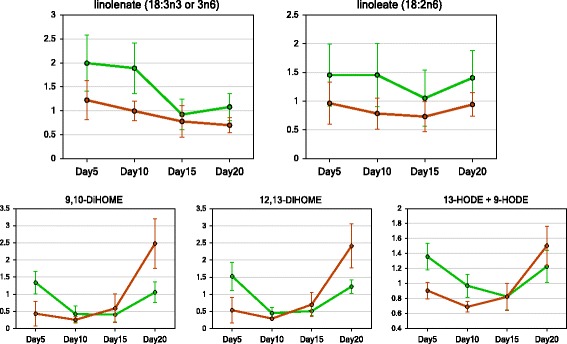
Dynamic changes of oxylipins in translucent (*green line*) and chalky (*dark red line*) endosperms during caryopses development. 9,10-DiHOME, (12Z)-9,10-Dihydroxyoctadec-12-enoic acid; 12,13-DiHOME, (9Z)-12,13-Dihydroxyoctadec-9-enoic acid; 9-HODE, 9-Hydroxyoctadecadienoic acid; 13-HODE, 13-Hydroxyoctadecadienoic acid. Day 5, 10, 15, and 20, days after anthesis

While the vast majority of lipids decreased over time in seed development, several oxylipins were among the few compounds increasing most rapidly during the late phases. The kinetic pattern for 9,10-diHOME, 12,13-id-HOME, and 13-HODE+9-HODE all showed very similar patterns, being higher at the outset but then declined during the mid-phase of development, and then spiked at the last time point (Fig. 6). However this pattern was somewhat earlier for the chalky endosperm. They increased between 10 DAA and 15 DAA, followed by a greater increase at 20 DAA, but the upper translucent part was constant or declining between 10 DAA and 15 DAA, then only began to increase after 15 DAA. This suggests that chalky endosperm was shifted approximately 5 days earlier in the regulation of these compounds.

#### CPGECs

In general, CPGECs increased as seed development progressed (Additional file 1). Pantothenate in CoA metabolism was increased for both the chalky and translucent parts, with the former having relatively higher level. In nicotinate and nicotinamide metabolism, nicotinamide adenine dinucleotide (NAD^+^) was slightly decreased in the chalky part on 5 DAA, while nicotinate and trigonelline (N’-methylnicotinate) were elevated during seed development. Phosphate decreased for both parts, and it was lower on 5 DAA and 10 DAA whereas slightly higher on 20 DAA in the chalky endosperm.

There were hints of differences in the oxidative stress environments for the two parts. Chalky endosperm showed higher levels of ascorbate, suggesting a less oxidative environment. Consistent with this, there were lower levels of two ascorbate catabolites, dehydroascorbate and threonate. In addition, amphiphilic antioxidants alpha-tocopherol and gamma-tocopherol were up-regulated on 20 DAA, but they were lower on 5 DAA and 10 DAA.

#### Purine and pyrimidine metabolism

Except adenine, adenosine, allantoin, guanosine-2′,3′-cyclic monophosphate, and uridine, the 17 detected metabolites in nucleic acid metabolism were all reduced for both parts during the four sampling times (Additional file 1). Compared with the translucent endosperm, chalky part had higher level of adenosine-2′,3′-cyclic monophosphate and 5′-GMP but lower level of adenosine, allantoin, and inosine.

#### Peptide

The majority of peptides were decreased for both parts during the four sampling times, and the chalky endosperm showed lower levels (Additional file 1). Interestingly, the dipaptide threonylphenylalanine was increased markedly in the chalky parts, in particular on 15 DAA and 20 DAA.

#### Phytohormones

Two phytohormones, ABA and IAA, as well as two compounds associated with physiological signaling in plants, serotonin and GABA, were detected (Fig. 4; Additional file 1). While the changes were not dramatic, chalky endosperm had lower ABA (although variable) early in seed development, while translucent part started higher and decreased over time. At the last time point ABA levels were essentially the same in both lines. IAA was similar for both lines, being slightly higher in chalky endosperm on 15 DAA, and showed an increase over time.

#### Secondary metabolites

Benzenoid 4-hydroxybenzoate decreased in the chalky and translucent part as seed development progressed, and it was relatively higher in the chalky endosperm on 20 DAA (Additional file 1). Phenylpropanoids indentified were decreased during seed development, with the exception that coumarate did changed significantly. In addition, ferulate, sinapate, and coumaroylquinate tended to be higher in the chalky endosperm on 15 DAA and 20 DAA. Terpenoid squalene was increased for both parts, and the chalky part was lower on 10 DAA but higher on 15 DAA.

## Discussion

### The notched-belly mutant

Grain chalkiness is a complex trait, controlled by multiple genes and varying with growing environments. So far, omics approaches have been employed to explore genes or biochemical pathways responsible for chalkiness [2, 14, 21], and our knowledge concerning chalkiness has been enlarged. However, only few QTLs or genes have been identified and functionally analyzed, and the metabolic mechanisms underlying chalkiness are still imperfectly understood.

We obtained a notched-belly mutant with high ratio of white-belly grains. The notched line can be seen on 5 DAA, possibly due to the restriction of the hulls on caryopsis elongation (Fig. 1). The constriction by bending alters the morphology of the caryposis, resulting in different shape of the upper and lower part, as shown in Fig. 1. It is worth studying if this change in morphology should cause disruptions in starch metabolism, granule formation and thereby chalkiness. Future studies on the loading routes of sucrose along the vertical axis of the notched belly seed versus the non-notched parent line are indispensible for clarifying the mechanism of this particular mutant.

The notched-line divided the endosperm into two separate compartments, with the upper part being translucent whereas the bottom being mainly opaque on about 20 DAA. These two parts of grain develop to mature independently, as they are separately supplied with assimilates from source organs through vascular bundles on the dorsal side. Thus the formation of the notched-line makes possible the chasing of the process of chalky tissue formation. Using the upper part as control, we compared the changes in metabolic profiles between the translucent and chalky part. Intriguingly, this comparison was made within the same genetic background, and is different from those performed either between varieties or between chalky grains and perfect grains for a given variety. Therefore, the findings of biochemical pathways such as carbohydrate metabolism, S-containing amino acid metabolism, and ROS scavenging are of relevance to chalkiness formation.

It is obvious that the position within grains contributes to difference in the occurrence of chalkiness between the two parts. Because of the notched-line, the upper endosperm is entirely isolated from the embryo, and thus is hardly affected by it. On the contrary, the bottom part is attached to the embryo, and is subjected to be influenced by the embryo. Thus the difference between the upper and lower parts was mainly associated with the compound effect of the chalkiness and the location within the grain (the effect of the embryo), as also discussed in our previous works on matured grains [14, 22]. Because it is impossible to recognize perfect grains or chalky grains at early stage, the compound effect of chalkiness and embryo can not be dissected at present time. Therefore the findings of this study have limitations when explaining the mechanisms underlying grain chalkiness, and the role of embryo in the formation of grain chalkiness needs to be thorough examined.

### Sensing and regulating C and N status

Starch and protein, the products of C and N metabolism, are major factors affecting rice grain quality. Grain chalkiness is the result of poor filling of starch granules or imbalances in the subtle adjustment to starch degradation pathway triggered under stress [2]. In addition, decrease in biosynthesis of proteins also plays crucial roles, as supported by the characterization of the recently cloned *Chalk5* [6] and the microscopic observation of chalky tissue [1]. C and N metabolism are therefore the key pathways toward chalkiness formation, and a comprehensive understanding of them is central to the study on grain chalkiness. However, the mechanism by which plants sense signals relating to C and N status and subsequently regulating them still remains open.

In the present study, we detected several key metabolites involved in sensing and regulating carbon and nitrogen status. The chalky part contained lower sucrose content, as was accompanied by decrease in Glu and Gln, while Asp and Asn, carrying more N atoms per C, were increased on 15 DAA and 5 DAA, respectively. The inverse changing patterns of Glu/Gln and Asp/Asn indicate that the C skeletons for biosynthesis of amino acids may be limited in the chalky part so that it uses a more economical compound to transport N. Supporting the argument above, Arg, with a high N:C ratio (4:6) and serving as a main N storage compound, was up-regulated in the chalky endosperm. This was partially due to higher levels of its precursors, N-acetylglutamate and citulline. In addition, Arg plays a major metabolic role in seed maturation and germination, phloem and xylem transport, and accumulates under stress conditions [23].

It has been suggested that GABA, a four-C nonprotein amino acid, constitutes a readily accessible nontoxic reserve of C and N for amino acid metabolism and tricarboxylic acid (TCA) cycle activity, which is of particular relevance during stress events. In Arabidopsis, GABA shunt metabolic pathway that converts Glu to GABA has been shown to have a profound effect on the C-N balance of the seed [24]. Our results showed that chalky endosperm contained significantly lower GABA during seed development, and its role in chalkiness formation merits further investigation.

### Pathways involved in chalkiness formation

#### S-containing amino acids

**Fig. 7:**
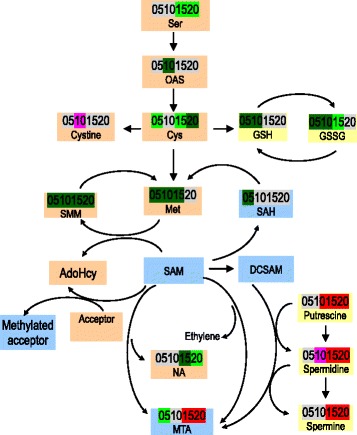
S-containing amino acids and their derivatives of the upper translucent part and the bottom chalky part. 05, 10, 15, and 20, days after anthesis. Refer to Additional file 1: Table S1 for detailed information of the changes of these metabolites

Sulfur is essential for synthesize Cys, Met, and other essential and secondary metabolites like MTA, SAM, and SMM [25]. Cys has a multitude of functions due to the chemical properties of reduced sulfur. Sulfide is a strong nucleophile, making it a highly reactive mediator of redox reactions. Cysteine is the donor of sulfide for all of these compounds in all cells. MTA is the common by-product of polyamines (PAs), nicotianamine (NA), and ethylene biosynthesis [26]. SAM plays numerous roles of being the major methyl-group donor in transmethylation reactions [27]. SMM, a methionine derivative, has been proposed to be an important long-distance transport form of reduced S [28]. Regardless of the increase in MTA, the present study shows reduction in the chalky endosperm of the majority of S-containing amino acids and their derivatives including Cys, Met, SMM, MetO, SAH, and OAS (Fig. 7). This reduction was accompanied by marked changes in the byproducts, the PAs and NA, indicating the role of S transport, assimilation, and its downstream metabolism in chalkiness formation.

#### ROS scavenging

**Fig. 8:**
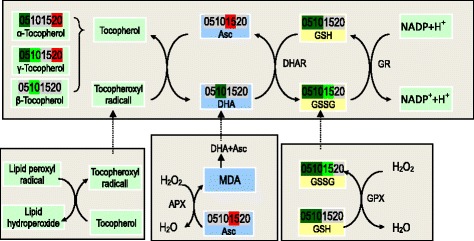
ROS scavenging network of the α-tocopherol-ascorbate-glutathione triad of the upper translucent part and the bottom chalky part. 05, 10, 15, and 20, days after anthesis. *Red* and *green* indicate *p* ≤ 0.05. Refer to Additional file 1: Table S1 for detailed information of the changes of these metabolites. *APX* ascorbate peroxidase, *Asc* ascorbate, *DHA* dehydroascorbate, *DHAR* dehydroascorbate reductase, *GPX* glutathione peroxidase, *GR* glutathione reductase, *GSH* reduced glutathione, *GSSG* oxidized glutathione, *MDAR* monodehydroascorbate reductase, *MDHA* monodehydroascorbate

The overproduction of ROS is likely to result in damage to proteins, lipids, carbohydrates and DNA in plant cells, in particular under abiotic stresses including drought, high temperature, and chemical toxicity [29]. ROS can be scavenged by non-enzymatic low molecular weight metabolites like ascorbate, glutathione and α-tocopherol, which form an important part of abiotic stress response [30]. Liu et al. [21] suggested that glutathione-S-transferase, glyoxalase, lipoxygenase-5, and thioredoxin involving in ROS scavenging may account for the formation of grain chalkiness. Using iTRAQ, Lin et al. [14] identified four proteins, catalase isozyme B, thioredoxin O, glutaredoxin-C8, and peroxidase 2, which were associated with ROS-scavenging mechanism of rice chalky endosperm. The current study shows lower levels of GSSG, GSH and 5-OP (Fig. 8). Consistent with this, higher levels of ascorbate and amphiphilic antioxidants α-tocopherol and γ-tocopherol were noted in the chalky endosperm, as was coined with lower levels of two ascorbate catabolites, dehydroascorbate and threonate. In addition, other metabolites with antioxidant activities such as PAs (Put, Spd, and Spm) were all up-regulated. Collectively, these findings suggest a less oxidative environment in the chalky endosperm.

#### Osmoregulation

The accumulation of osmolytes is the common strategy adopted by plants to combat the environmental stresses. Among them, betaine has strong osmoprotectant properties and confers tolerance to salinity and drought [31]. Trehalose has also been proven useful to improve abiotic stress tolerance [32]. The potential role of raffinose in stress tolerance has been intensively studied in seeds, particularly with respect to desiccation tolerance and longevity in the dehydrated state [33]. Three polyamines namely Put, Spm, and Spd are considered as involved in variety of physiological response to abiotic stresses like salt and drought [34]. High temperature during grain filing stage causes high occurrence of chalkiness, and recent studies indicated a link between osmoregualation and chalkiness formation under heat stress. Wada et al. [35] assessed the effects of dry wind on chalky ring formation in a growth chamber. They concluded that osmotic adjustment plays a crucial role in the down-regulating of starch biosynthesis, which leads to chalky ring formation under short-term hot and dry wind conditions. In addition, magnetic resonance imaging of the early stage caryopses in the high-temperature condition revealed lower water content around the centre of the endosperm, where the chalky tissues occurs [36]. Osmolytes varied significantly between the chalky part and translucent part in this study. Although Pro was reduced, most of the osmolytes identified including betaines (glycine betaine, trigonelline, and stachydrine), trehalose, raffinose, and PAs were all increased in the chalky endosperm, suggesting that osmoregulation be triggered in the chalky endosperm.

#### Cell wall synthesis

Plant cells are surrounded by a cell wall that plays a key role in plant growth, structural integrity, and defense. Liu et al. [21] showed that down-regulation of genes responsible for cell wall synthesis (two cellulose synthase genes) and up-regulation of genes for cell wall hydrolysis (α-L-arabinofuranosidase and α-D-xylosidase) were linked with chalkiness occurrence. By contrast, previous proteomic survey of Lin et al. [14] demonstrated that five enzymes responsible for cell wall synthesis, including rhamnose biosynthetic enzyme, UDP-glucose 4-epimerase, UDP-glucose 6-dehydrogenase, UDP-arabinopyranose mutase, and UDP-glucuronic acid decarboxylase, were all up-regulated in the chalky endosperm of grains with white-belly. The cell wall is a complex and diverse structure that is mainly composed of polysaccharides. In the current study, we identified five precursors of cell wall (rhamnose, arabinose, glucose, gluconate, and xylose) that changed markedly between the chalky part and translucent part. Notably, they showed similar decreasing trend in the chalky endosperm, although arabinose did not varied significantly. Altogether, these studies indicate the relation of the cell wall synthesis with chalkiness formation.

#### Mineral ion homeostasis

Plants acquire minerals for sustaining growth and development. We identified several metabolites related to translocation and storage of minerals such as phytic acid, nicotianamine, and phytosphingolipids, which changed significantly between the chalky and translucent endosperms. Phytic acid is a nearly ubiquitous component of plant seeds and is usually the most abundant form of phosphate [37]. It has negatively charged phosphates, making it an efficient chelating agent of mineral cations like K, Mg, Fe, Ca and Zn. Nicotianamine is responsible for the translocation of Fe and Mn from leaf to the developing seeds [38]. The sphingolipids are a diverse class of membrane lipids that constitute up to 20 to 30% of the tonoplast and plasma membrane lipid in plants [39]. Although their roles are not well defined, recent studies indicate that they have critical functions such as playing an important role in mineral ion homoeostasis [40]. Phytic acid and its precursors were higher in the chalky endosperm, and this was accompanied by a simultaneous increase in raffinose. In soybean, a single, recessive mutation also confers a synchronous change of phytic acid and raffinose, which may be due to the fact that they share at least *myo*-inositol-1-phosphate and possibly free *myo*-inositol as a common intermediate [33]. In addition, nicotianamine and phytosphingosine were both down-regluated in the chalky endosperm. The results indicate different ability of mobilizing or storing the minerals between the chalky and translucent endosperm, which needs verification by analysis of the mineral composition.

## Conclusions

In summary, the notched-belly mutant is applicable to explore the physiological and molecular foundation of grain chalkiness, for it provides a high efficient comparison under the same genetic background that can minimize the influence of genotype and environment. Using this mutant, we observed a reduction in activity of C and N metabolism used for starch, protein, and cell wall synthesis in the chalky endosperm, which coincided with the disturbance of other pathways like osmoregulation, ROS scavenging, and ion homeostasis, implying a stressful condition (oxidative or osmostic) in the chalky part. It is still uncertain what triggers this metabolic shift. In addition, we cannot rule out the possibility that the differences in metabolite levels between the two sections are caused by some constriction between them due to the notched line; however, there is currently no evidence that this is the case. Clearly the development of chalkiness is a complex trait and this is corroborated by the metabolomic analysis in this study. An integrated approach using a combination of omics platforms and an examination of the physiology of the grain will be needed to understand this phenomenon fully.

## Methods

### Plant materials

Wuyujing3 had become a popular japonica rice cultivar in southern China with high eating quality since it was released in 1992 [41]. It is an inbred cultivar and not a hydrid or transgenetic cultivar. The seeds of Wuyujing3 were provided by the breeder of Wujin Academy of Agricultural Sciences, Jiangsu China. In 2007, about 2 kg seeds of Wuyujing3 were treated by 0.5% ethyl methane sulfonate, and then were sown in paddy field and mixed harvested as a mutant population. A notched-belly mutant with white-belly (DY1102) was identified from the M2 population [14]. It had high ratio of notched-belly grains, ranging from 66.58% (greenhouse) to 75.67% (ambient temperature) in 2014. Further, it had high occurrence of white-belly mainly on the bottom part, being 85.12% under greenhouse and 93.13% under high night temperature treatments (Table 1). Notably, grains on the middle primary rachis has higher ratio of white-belly, and were sampled for metabolic analysis.

In 2014, five plants of DY1102 were hand transplanted in a plastic pot filled with 15 kg clay soil, 30 cm in height and 34 cm in diameter. The soil contained 0.83 g/kg total N, 10.72 mg/kg available P, and 69.15 mg/kg exchangeable K. The basal fertilizers before transplanting were 1.0 g N, 1.2 g P_2_O_5_, and 0.9 g K_2_O/pot, and those for topdressing at the panicle initiation stage were 1.0 g N, 0.6 g P_2_O_5_, and 0.9 g K_2_O/pot. All the plants were grown under natural environment condition at seedling stage. Approximately 2–3 days before anthesis, 45 pots were transferred to a 28/23 °C plant chamber (12 h day–12 h night cycles; Model FYS-10; Hengyu, Nanjing, China) until maturity. This plant chamber has the light intensity above 75% of ambient light condition, and the relative humidity was maintained 70 ± 5% by a humidity regulator [42]. Flowering dates of caryopsis on middle primary rachis were marked. Sampling time points were 5, 10, 15, and 20 days after antheisis (DAA), conducted at 9:00 am. Grains were frozen by liquid nitrogen and then stored at −80 °C until analysis. Optical photos of developing grains were taken by stereomicroscope (Stemi 2000-C, Zeiss Jena GmbH, Germany) with digital camera (PC1145, Canon, Japan). For metabolomic analysis, the developing grains were dehusked, and the embryos were removed. Subsequently, the remaining endosperms were cut into upper tranlucent parts (Tr) and bottom chalky parts (Ch) along the notched line. Each sample was measured with six biological replicates.

### Metabolite profiling by multiple analytical platforms

Metabolite profiling was performed by SJTU-Metabolon Joint Metabolomics Laboratory (http://www.metabolon.com/) by the composite platforms: UPLC/MS/MS positive ion mode, UPLC/MS/MS negative ion mode, and GC/MS [16]. Briefly, the upper and lower parts of the endosperm were grounded into fine powders under liquid nitrogen condition. Metabolites in lyophilized powder (about 40 mg) were extracted using 400 μL of methanol with internal standards. The resulting extraction solution was divided into 3 parts and then freeze-dried. UPLC-MS/MS analysis included both positive and negative ion modes, the products of which were redissolved and then carried out on Waters Acquity UPLC (Milford, USA) coupled with a Thermo LTQ XL MS (Thermo Inc., Massachusetts, USA). For GC-MS analysis, samples were derivatized by bistrimethyl-silyltrifluoroacetamide (BSTFA) and analyzed on Thermo Ultra GC-ISQ platform equipped by an electron impact ionization system (Thermo Inc., Massachusetts, USA).

### Data analysis and construction of the metabolic atlas

Raw data of three analytical platforms were extracted, peak-identified and quality control processed using Metabolon Laboratory Information Management System. Metabolites were identified by automated comparison to reference library that was created using approximately 1500 authentic standards in Metabolon. Metabolites quantification, data normalization, and significant changed metabolites analysis were performed according to Lawton et al. [43] and Rao et al. [16]. Briefly, a data normalization step was conducted to correct variations due to instrument inter-day tuning differences. Metabolites quantitative values were derived from integrated raw detector counts of the mass spectrometers. To preserve all of the variation, all compounds of widely different raw data were normalized directly on a similar graphical scale, with the normalized intensities scaled by their median values for each compound (Additional file 1). Significance tests were performed by Welch’s two-sample *t*-test using the programs R. Metabolic pathways were determined by reference to databases Plant Metabolic Network (PMN; http://www.plantcyc.org/) and Kyoto Encyclopedia of Genes and Genomes pathway (KEGG; http://www.genome.jp/kegg/pathway.html) to construct the metabolic atlas.

## Additional file

Additional file 1: List of all metabolites identified and quantified by UPLC/MS/MS and GC/MS. (XLS 1325 kb)

## Abbreviations

5-OP: 5-oxoproline
C: Carbon
CPGECs: Cofactors, prosthetic groups, electron carriers
DAA: Day after anthesis
F6P: Fructose 6-phosphate
G1P: Glucose 1-phosphate
G3P: Glycerol-3-phophosphate
G6P: Glucose 6-phosphate
GABA: γ-aminobutyric acid
GSH: Reduced glutathione
GSSG: Oxidized glutathione
I6P: Inositol 6-phosphate
MetO: Methionine sulfoxide
MTA: 5′-methylthioadenosine
N: Nitrogen
NAG: N-acetylglutamate
OAS: O-acetylserine
ROS: Reactive oxygen species
SAH: S-adenosyl-homocysteine
SAM: S-adenosylmethionine
SMM: S-methylmethionine
TCA: Tricarboxylic acid
